# MEDICATION DISCREPANCIES AND COMMUNICATION ERRORS DURING NURSING HOME INTAKE: A MIXED-METHODS ANALYSIS

**DOI:** 10.1093/geroni/igz038.2680

**Published:** 2019-11-08

**Authors:** Mark E Patterson, Sandra Bollinger, Janice Foust

**Affiliations:** 1 University of Missouri-Kansas City School of Pharmacy, Kansas City, Missouri, United States; 2 Health Priorities, Inc., Cape Girardeau, Missouri, United States; 3 University of Massachusetts-Boston, Boston, Massachusetts, United States

## Abstract

Older adults experience high medication discrepancy rates during transitions from inpatient to nursing home settings. Dosage changes and multiple prescribers increases the risk of inaccurate handoffs and creates challenges for medication reconciliation at nursing home intake. Our objectives were to 1) Characterize medication discrepancies occurring at nursing home intake and 2) Identify resident and medication related factors associated with medication discrepancies. Demographics, comorbidities, medications, discrepancy types and location were prospectively collected over 9-months. Chi-square tests were used to determine factors associated with discrepancies. A focus group of nurse practitioners, pharmacists, and administrators from four long-term care facilities was convened to discuss medication reconciliation challenges at resident intake. Thematic analysis was used to determine key themes. 22%, 12%, and 3% of residents experienced one, 2 to 5, or six or more discrepancies, respectively. The most prevalent discrepancies were omission (34%), frequency (20%), and therapeutic duplication (13%) occurring in analgesics, respiratory and genitourinary medications. 44% of discrepancies occurred between nursing homes and hospitals and 39% involved the community pharmacy. The most significant risk factors for discrepancies included age over 70, Charlson comorbidity indices over 7, readmission to nursing homes, or the prescribing of at least 17 medications. Staff faced challenges of delayed and/or inaccurate data, incompatible documentation forms and inefficient workflows for resolving discrepancies. Residents at greatest risk for medication discrepancies require additional attention during admission medication reconciliation to prevent errors. Nursing home intake and medication reconciliation workflow needs to be improved with data sharing technology to increase accuracy and efficiency.

